# Neuro Emotional Technique for the treatment of trigger point sensitivity in chronic neck pain sufferers: A controlled clinical trial

**DOI:** 10.1186/1746-1340-16-4

**Published:** 2008-05-21

**Authors:** Peter Bablis, Henry Pollard, Rod Bonello

**Affiliations:** 1 Macquarie Injury Management Group, Macquarie University, Sydney, Australia; 2 Director of Research, ONE Research Foundation, Encinitas, California, USA

## Abstract

**Background:**

Trigger points have been shown to be active in many myofascial pain syndromes. Treatment of trigger point pain and dysfunction may be explained through the mechanisms of central and peripheral paradigms. This study aimed to investigate whether the mind/body treatment of Neuro Emotional Technique (NET) could significantly relieve pain sensitivity of trigger points presenting in a cohort of chronic neck pain sufferers.

**Methods:**

Sixty participants presenting to a private chiropractic clinic with chronic cervical pain as their primary complaint were sequentially allocated into treatment and control groups. Participants in the treatment group received a short course of Neuro Emotional Technique that consists of muscle testing, general semantics and Traditional Chinese Medicine. The control group received a sham NET protocol. Outcome measurements included pain assessment utilizing a visual analog scale and a pressure gauge algometer. Pain sensitivity was measured at four trigger point locations: suboccipital region (S); levator scapulae region (LS); sternocleidomastoid region (SCM) and temporomandibular region (TMJ). For each outcome measurement and each trigger point, we calculated the change in measurement between pre- and post- treatment. We then examined the relationships between these measurement changes and six independent variables (i.e. treatment group and the above five additional participant variables) using forward stepwise General Linear Model.

**Results:**

The visual analog scale (0 to 10) had an improvement of 7.6 at S, 7.2 at LS, 7.5 at SCM and 7.1 at the TMJ in the treatment group compared with no improvement of at S, and an improvement of 0.04 at LS, 0.1 at SCM and 0.1 at the TMJ point in the control group, (P < 0.001).

**Conclusion:**

After a short course of NET treatment, measurements of visual analog scale and pressure algometer recordings of four trigger point locations in a cohort of chronic neck pain sufferers were significantly improved when compared to a control group which received a sham protocol of NET. Chronic neck pain sufferers may benefit from NET treatment in the relief of trigger point sensitivity. Further research including long-term randomised controlled trials for the effect of NET on chronic neck pain, and other chronic pain syndromes are recommended.

**Trial Registration:**

This trial has been registered and allocated the Australian Clinical Trials Registry (ACTR) number ACTRN012607000358448. The ACTR has met the requirements of the ICMJE's trials registration policy and is an ICMJE acceptable registry.

## Background

Trigger points have been defined as discrete, hyperirritable foci usually located within a taut band of skeletal muscle [1]. The point is a well-circumscribed area in which pressure produces a characteristic referred pain, tenderness and autonomic phenomena [1]. Trigger points are considered an essential defining part of the myofascial pain syndrome, in which widespread or regional muscular pain is a cause of musculoskeletal dysfunction [2], as well as being associated with hyperalgesia, restriction of daily function or psychological disturbance [3]. Upon clinical presentation, trigger points are classified depending on certain characteristics. An active trigger point is defined as one with spontaneous pain, or pain in response to movement. It is tender on palpation, and may present with a referral pattern of pain, not at the site of the trigger point origin. A latent trigger point is a sensitive spot that causes pain or discomfort only in response to compression. Trigger points are reported to occur more frequently in cases of mechanical neck pain than in matched controls [4]. Patients may only become aware of pain when pressure is applied to a muscular point of restriction or weakness.

The pathogenesis of trigger points is not clear, but it is believed they arise from more than one cause [5]. Fischer [5] has suggested that trigger points are due to the sensitisation of nerves and the tenderness results from the decrease in the pain pressure threshold. He further opines that the tissue damage associated with injury causes the release of inflammatory products that increase the sensitivity of the nerve to stimulation. These substances include bradykinins, 5-HT and prostaglandins, though a recent study found tender points in the trapezius muscle of patients with tension-type headache were not sites of ongoing inflammation [6]. Trigger points are also thought to arise from acute trauma or repetitive microtrauma, such as lack of exercise, poor nutrition, postural imbalances, vitamin deficiencies, sleep disturbances and joint problems [7]. One study suggests overloading of muscle fibres may lead to involuntary shortening, oxygen and vitamin deficiencies and increased metabolic demand on local tissues [8], and trigger points have been suggested as decreasing the extensibility and contractile efficiency of muscles, and possibly causing muscle fatigue [9]. This is yet to be confirmed by research.

Trigger points have been shown to be active in fibromyalgia [10,11], as well as somatic tenderness secondary to visceral dysfunction [2], migraine and other forms of non-pathological headache [12], shoulder [13] neck [14] and back pain [15]. Specifically, Rosomoff and co-workers [15] demonstrated that approximately 97% of persons with chronic intractable pain have trigger points, and of these, 45% have a non-dermatomal referred pain. Furthermore, Rosomoff's team demonstrated that 100% of neck pain sufferers possessed the presence of trigger points and almost 53% of them had non-dermatomal referral [15]. However, it is worthy of note that no evidence describes the prevalence of trigger points of the neck and face in a normal population. Indirect evidence presented in the equine model suggests there to be significant differences between active trigger points and control points [16].

The diagnosis of a trigger point involves physical examination by an experienced therapist using a set of cardinal signs (Table 1) [1]. There have been many studies focused on the assessment of the reliability of detecting trigger points. Lew et al. [17] found that both inter and intra-rater reliability, using two highly trained examiners was poor, while Gerwin et al [18] found that extensive training of four clinicians together resulted in improved reliability for the identification of trigger points. Reeves et al. [19] demonstrated a moderate degree of intra and inter examiner reliability in determining the location of trigger points. In older studies values ranged from r = 0.68 to r = 0.86 [19]. In a study by Delaney and McKee [20], interclass correlation co-efficient (ICC) revealed inter-rater reliability to be high (values ranged from ICC = 0.82 to ICC = 0.92), and intra-rater reliabilities to be high (values ranged from ICC = 0.80 to ICC = 0.91) for the use of a pressure threshold meter in measuring trigger point sensitivity.

**Table 1:** The Cardinal Signs of a Trigger Point (adapted from Simons, Travell and Simons [1]).

**Cardinal Signs of a Trigger Point**	
-	Presence of a taut band in the target muscle
-	A nodular point of tenderness
-	A jump sign: Patient reacts to the application of digital pressure to the taut band or nodular point
-	Referral of pain on the application of pressure to the taut band or nodule

In both clinical and experimental practice, a device such as the pressure algometer would be of great value for reliable quantification of trigger point sensitivities, once manually located. Fischer [5] demonstrated that the use of algometry in the detection of trigger points was a reliable procedure. He assessed the pressure threshold of deep tenderness in soft tissues, before and after various forms of treatment such as physiotherapy and drug therapy. In addition, Reeves et al. [19] reviewed studies that demonstrated the reliability of the pressure algometer. He found that an experimenter was able to reliably obtain similar measurements on two occasions, as well as produce similar scores to independent experimenters. He also noted that agreement was found between two experimenters when locating unmarked trigger points and measuring their sensitivity, but did stress the importance that experimenters were experienced and trained. In patients who present to manual therapists, the use of algometry can be used to reliably quantify the tenderness associated with a trigger point and can be used to diagnose their location as well as to qualify the degree of pressure sensitivity.

Trigger points are potential outcomes of dysfunction in a region, and conventional treatment is based around the release of this taut band of skeletal muscle. Manual therapy [21], chiropractic treatment [1,22], electric therapy [23], local anaesthetic [24] and active therapy [25] have all been claimed to provide relief of trigger point sensitivity. Injection therapies involved the use of local anaesthetic and saline, while it is postulated that massage and myofascial release aim to increase local circulation, improve mobility and relieve subcutaneous tightness. Furthermore, the presence of trigger points has been frequently associated with signs and symptoms in addition to pain [26], and these syndromes may be in found in disorders associated with chronic psychosocial factors [27]. Whilst it is likely the pathogenesis has at least a partly central mechanism, most approaches to the management of the trigger point phenomenon utilise only peripheral approaches to the points themselves.

Therapy for trigger points requires an approach that enhances the central inhibition through pharmacological or behavioural techniques, and reduces the peripheral inputs to the maintenance of the reflexes by utilising physical therapies such as exercise [28], needling and digital pressure [29]. Offenbacher & Stucki [30] have also suggested that a combined approach to therapy would be warranted for patients exhibiting myofascial (as well as other) symptoms in conditions such as fibromyalgia. It was the specific aim of this research to investigate whether a new mind body technique called Neuro Emotional Technique (NET) could significantly relieve pain sensitivity of trigger points presenting in a cohort of neck pain sufferers.

This study investigated the effects of Neuro Emotional Technique (NET) on the sensitivity of trigger points presenting in regions of the neck including the suboccipital region, levator scapulae region, sternocleidomastoid insertion region and temporomandibular region, in a cohort of chronic neck pain sufferers. The results of the study could provide useful information or the treatment of cervical pain and related psychosocial problems.

## Methods

This study received ethics approval from the Macquarie University Ethics Committee, reference number: HE24AUG2007-D05403.

### Participants

This research was performed in a private practice setting in Sydney, Australia. A convenience sample of sixty consecutive participants was recruited from new patients presenting during the period between February 2005 and June 2005. Every third consecutive chronic neck pain participant was allocated to a blinded control group to eliminate selection bias. This protocol resulted in 40 participants allocated to the treatment group and 20 allocated to the control group. Sequential allocation was concealed from the participants. Participants provided informed written consent prior to participating in the project.

All participants presenting with chronic cervical pain (greater than 3 months duration) as their primary complaint were invited to participate. Cervical pain was defined as pain located from a horizontal line drawn at the level of the 1^st ^thoracic vertebrae to a horizontal line drawn at the level nuchal line of the occiput, and laterally to the lateral border of the trapezius muscles. Those participants who did not have neck pain and headache, or have acute cervical pain were excluded from the study, as were participants under 18 years of age, or had undergone recent surgery or were suffering any concurrent pathology.

### Pre-Treatment Protocol

**Figure 1:**
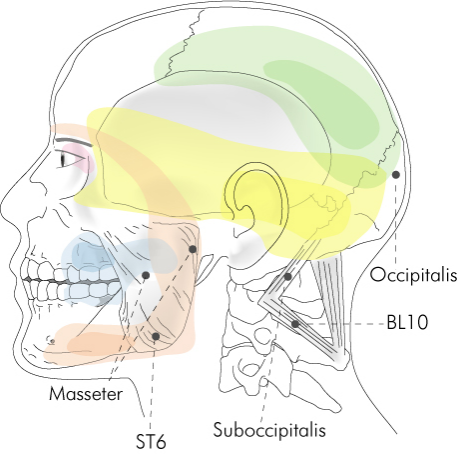
Suboccipital and temporomandibular trigger point region.

**Figure 2:**
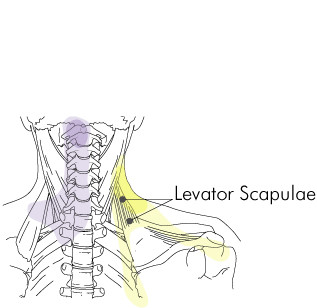
Levator scapulae insertion trigger point region.

**Figure 3:**
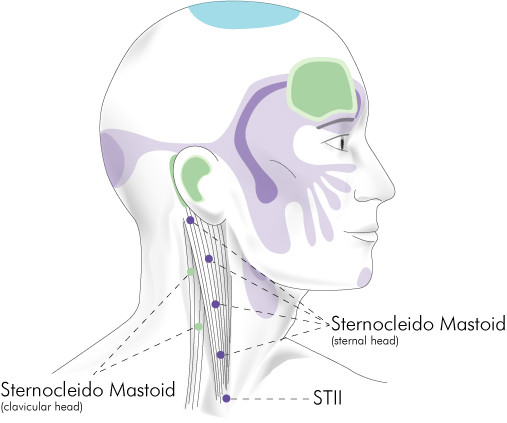
Sternocleidomastoid insertion trigger point region.

All participants underwent a standard patient evaluation that included an interview, a questionnaire and a standard physical examination. This provided information for each participant in terms of age (years), sex, cause of injury (i.e. motor vehicle accident), and duration of pain (months). In addition, participants were evaluated for the presence of cardinal signs of a trigger point (See Table 1) at four specific areas. The four areas of trigger points specifically targeted were the belly of the suboccipital (S) muscle, levator scapulae (LS) insertion, sternocleidomastoid (SCM) insertion and the belly of the masseter muscle (referred to as the temporomandibular (TMJ) region) (Figures 1, 2, 3).

The patients' perceived pain levels were assessed via a 100 mm visual analog scale (VAS) while the examiner palpated and verified the existence of the trigger point. Participants were asked to score the intensity of the pain using a metal slider with a graduated 10-centimetre rule. Participants were asked to rate their pain with zero (0) being no pain and ten (10) being the worst pain they could imagine by sliding the ruler to the point that represented their pain on the scale 0 to 10. Gallagher reports a 13 mm difference on the VAS represents the smallest measurable change in pain severity that is clinically important [31].

Furthermore, a pressure gauge algometer (PGA: Pain Diagnostics & Thermography Inc, 17 Wooley Lane East, Great Neck, NY 11021, USA) was used on the location of the trigger point to assess the amount of pressure the patient could sustain before the patient registered the pressure as being painful. These assessments were performed before and after the intervention. The pain threshold meter or PGA consists of a rubber disc with a surface of exactly 1 cm^2 ^that is attached to a force (pressure) gauge. The gauge is calibrated in kilograms and pounds. According to Smyth [32], the use of the device with a 1 cm^2 ^disc rather than other larger sizes at its tip has been called more suitable for the assessment of deep muscle trigger points.

**Figure 4:**
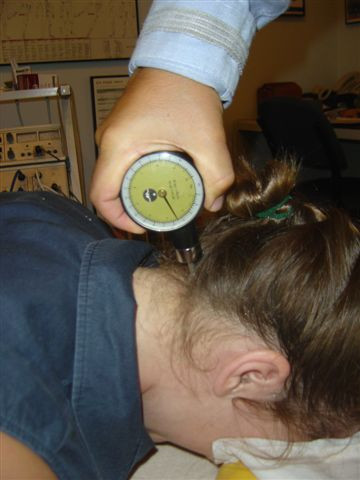
Suboccipital region pressure gauge algometer application.

**Figure 5:**
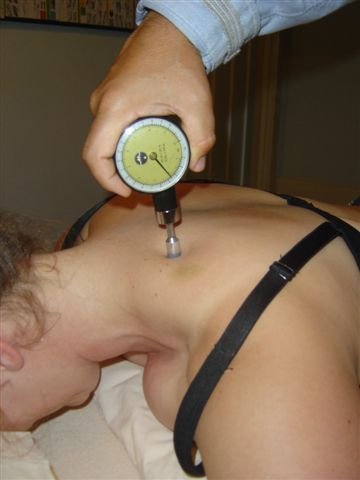
Levator scapulae insertion region pressure gauge algometer application.

**Figure 6:**
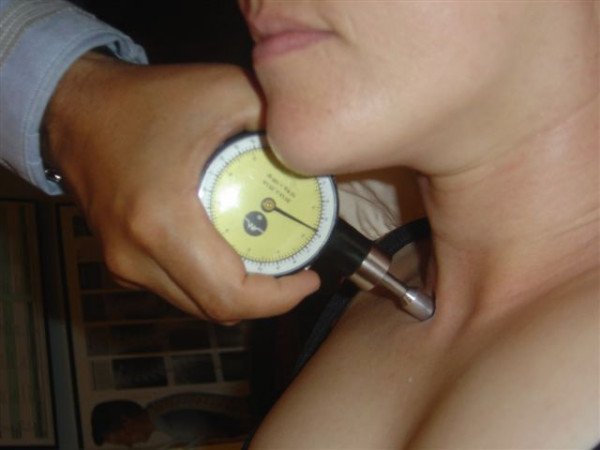
Sternocleidomastoid insertion region pressure gauge algometer application.

**Figure 7:**
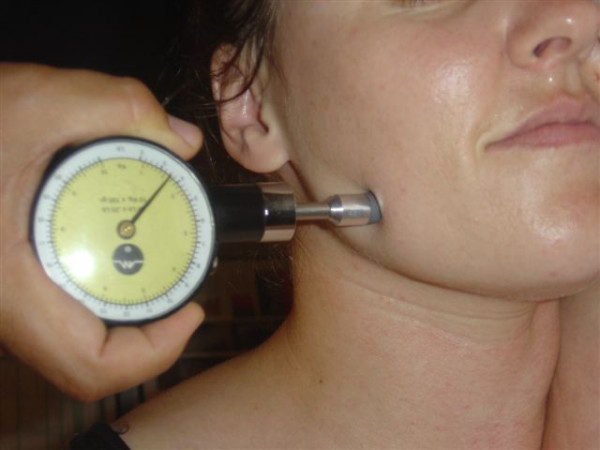
Temporomandibular region pressure gauge algometer application.

The participants had the use of the PGA explained to them using the protocol of Fischer [33], and also the location of the pain. Following the explanation, the point of maximum tenderness was determined and marked with indelible ink. Once marked, the pressure threshold was determined using a control point in a non- painful muscle in an unrelated area. The control point was taken as the equivalent point on the opposite side of the body if not tender; if tender another soft tissue non-tender point was identified. Once determined, the practitioner guided the tip of the gauge between the finger and the thumb to avoid slipping by rounded contours. It was applied perpendicular to the long axis of the structure on which it was placed (Figures 4, 5, 6, 7).

In addition, all participants were asked to rate the intensity of the pain at the trigger point location on the application of progressively increasing increments of pressure (0.45 kg/2.54 cm^2 ^(1 lb/inch ^2^) at 0.45 kg (1 lb) every one second [34]). The practitioner used a PGA to score the pain. The PGA measures the depth of depression of the muscle during the application of pressure through the device by the practitioner. Utilising this protocol, the practitioner was able to determine the gross amount of depression for a given pain score [34].

**Figure 8:**
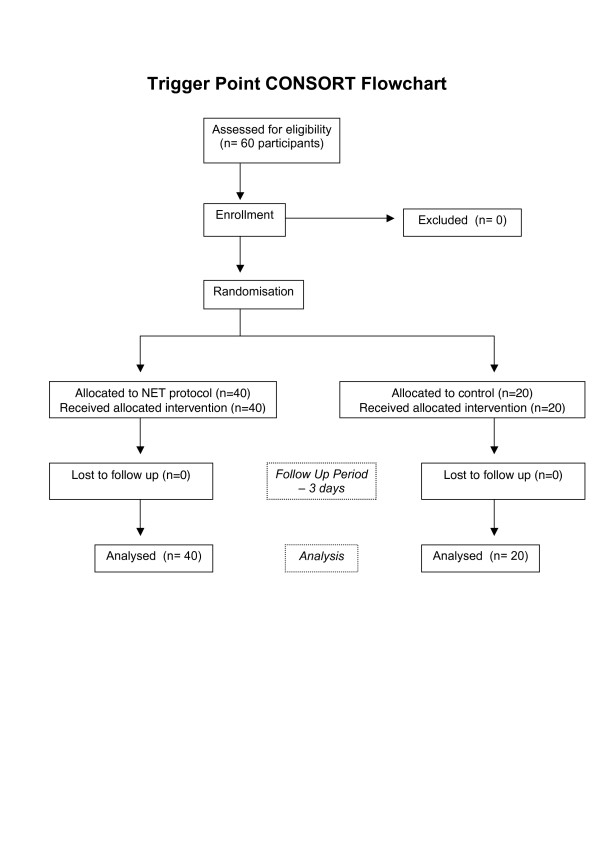
Trigger Point study – CONSORT Flowchart.

After participants were assessed for the cardinal signs of a trigger point (Table 1) both groups received their assigned intervention protocol. The control group received sham NET protocol, whilst the treatment group received NET protocol. Participants returned for re-assessment three days post intervention (See Figure 8).

### NET Treatment Protocol

**Figure 9:**
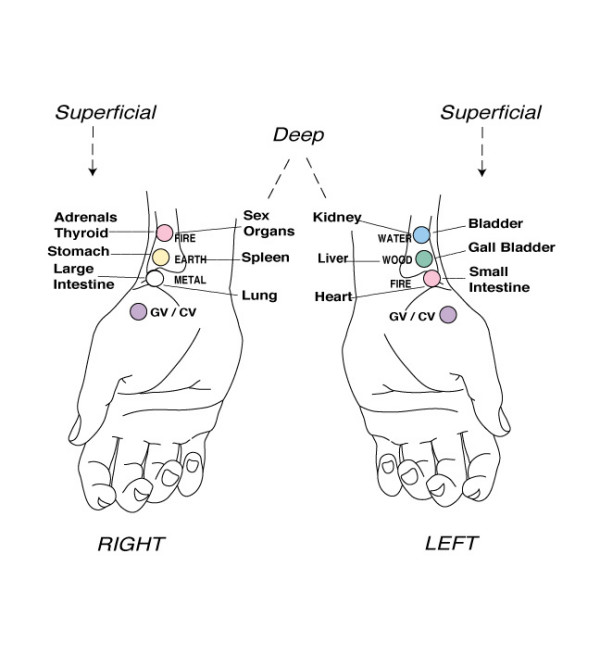
Acupuncture pulse points used at the wrist. From Walker 1996 [34] (Used with permission).

After assessment, participants in the treatment group underwent the NET protocol as outlined by Walker [35]. The detailed first 12 steps of the NET protocol used in this study are shown in Additional File 1. Neuro Emotional Technique, often described as a complementary and alternative medicine modality, was administered during the study as a technique designed to incorporate central and peripheral components to alleviate the effects of distressing stimuli [36]. Developed by Walker [35], NET has been described as a 15 step, multi-modal intervention that incorporates principles of several health disciplines, including cognitive behavioural psychology, traditional Chinese medicine pulse assessment, and a feedback technique called the muscle test [37]. A major goal of NET is to achieve a reversal (or extinction) of classically conditioned distressing emotional responses to trauma related stimuli, stimuli that have the characteristic ability to reproduce or augment pain and other signs of disease without the original stressor(s) being present. This objective is similar to treatments of standard cognitive behavioural therapy for traumatic stress, such as exposure therapy [36]. NET differs from such treatments in that it supposedly engages the energy system as it is conceived in the traditional Chinese medical model [38]. This entails the patients touching the relevant pulse point on the wrist that is determined to be involved in the body's stress reaction to the given stimuli (Fig 9.) Using principles of traditional Chinese five-element theory [39], the therapist helps the patient identify the particular pulse point using an application of major energy channels, or 'meridians', that contain specific emotional qualities. In the NET framework it is thought that the engagement of the body's energy system in the cognitive-emotional processing of an event facilitates a resolution to the event [40]. NET aims to help patients become less physiologically reactive to distressing stimuli and to become more competent in choosing alternative responses. NET is intended to be a brief, time-limited intervention. Several recent publications have discussed the use of NET for conditions such as hypothyroidism [41] and polycystic ovarian related infertility [42]. In the only clinical trial, investigators demonstrated a significant decrease in phobic symptoms following a brief course (2–3 visits) of a variation of NET [43,44].

Participants were asked to return 3 days later for assessment of the pain level using the VAS and to determine the status of the four cardinal signs of a trigger point using the PGA.

### Control Treatment Protocol

After the initial assessment, participants in the control group underwent the sham NET protocol. This included an information session, painless palpation of trigger points and administration of a sham NET protocol. Participants were asked to return 3 days later for assessment of the pain level using the VAS and to determine the status of the four cardinal signs of a trigger point using the PGA.

The assessments were performed by a chiropractor (number one) and the treatment was provided by another chiropractor (number two). The results of the study were tabulated by a third chiropractor. Chiropractor number one was unaware of the assignment of subjects to groups.

### Statistical Analysis

The results were analysed using parametric statistics because, within each treatment group, each of the dependant variables exhibited uni-modal frequency distributions that did not differ significantly from normal distributions. In all cases the threshold probability for significance was 0.05.

Several different statistical methods were adopted. Data analysis of group participation was performed using analysis of variance (ANOVA). The experimental design for this project is split-plot, with patients allocated to treatments and assessments made on each patient pre and post treatment. As only two assessments were involved, the split-plot analysis is equivalent to a Students t-test of differences (pre-post for VAS scores and post-pre for PGA measurements. We used GenStat (Payne et al, 2008) for these analyses, with the package testing for treatment variances automatically before testing for means.

## Results

### Participants

The average age for participants was 44.1 yrs old (S.D. = 11.7 yrs), with 56% female participants in the treatment group and 55% in the control group. The average duration of pain for all participants was 21.0 months (S.D. = 20.5 months). The severity of pain (an average of VAS scores across trigger points) for patients arriving at the clinic was 8.8 (S.D. = 0.51). There were no significant differences between the two groups for any of these variables (see Table 2 for details).

**Table 2:** Baseline comparisons of control and treated groups of patients

	Control (n = 20)		Treatment (n = 40)		
	Mean	SEM^1^	Mean	SEM	P for comparison

Age (years)	41.2	2.37	45.6	1.90	0.180
Time with pain (months)	19.5	3.59	21.7	3.57	0.700
Severity of trigger point pain on presentation (VAS)	8.9	0.08	8.8	0.09	0.374

SEM: Standard error of mean

### Comparison of control and treatment groups

**Figure 10:**
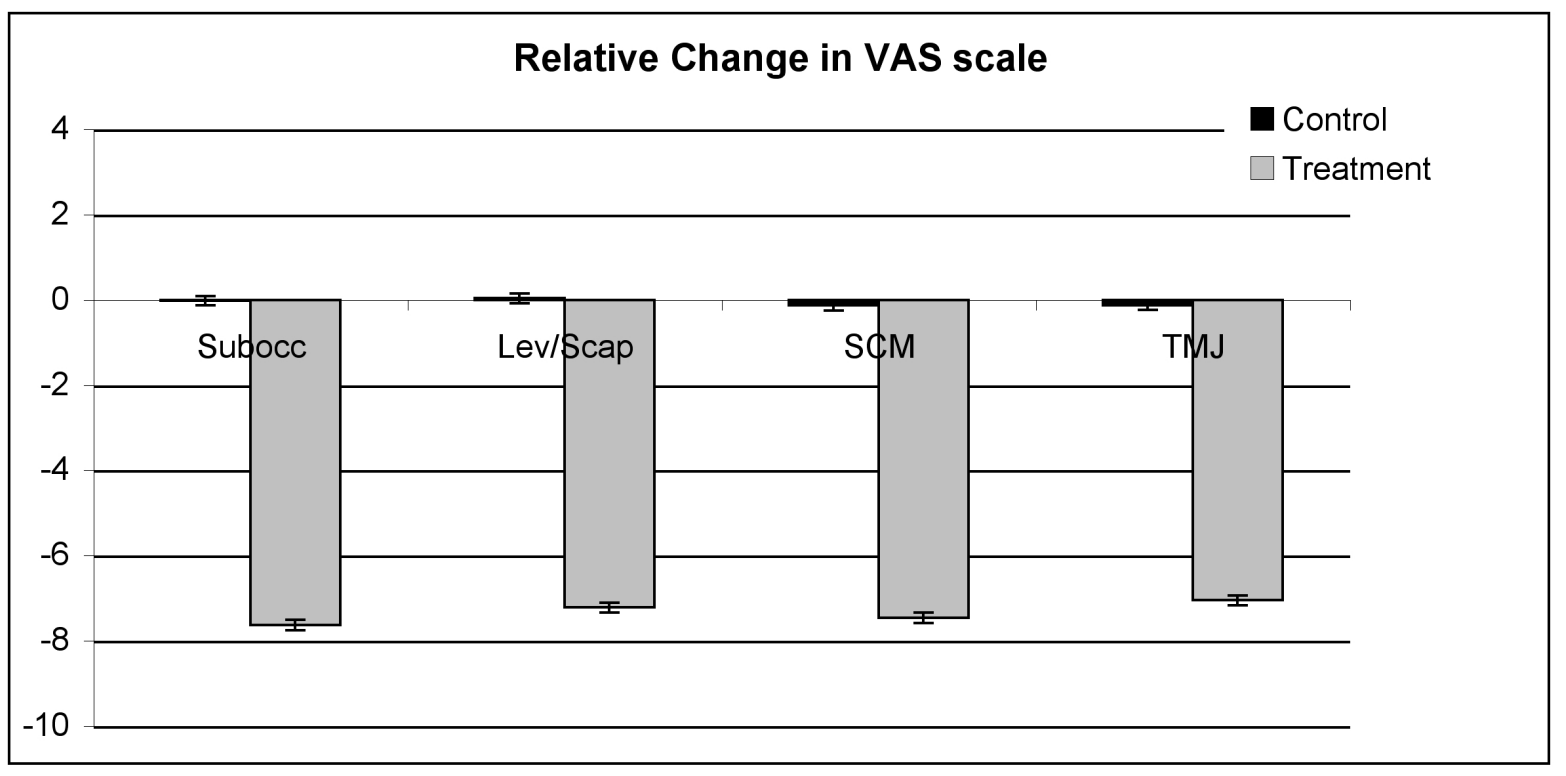
**Relative changes to visual analog scale scores**. (VAS: visual analog scale; PGA: pressure gauge algometer readings; Subocc: suboccipital trigger point location; Lev/Scap: levator scapulae insertion trigger point location; SCM: sternocleidomastoid trigger point location; TMJ: temporomandibular trigger point location).

**Figure 11:**
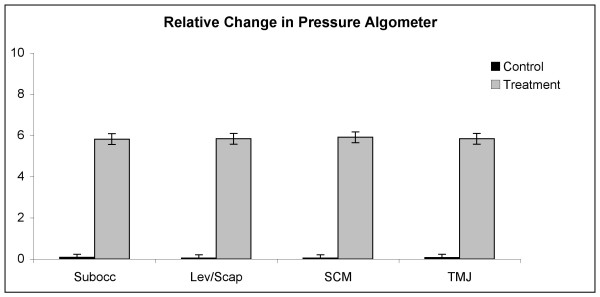
**Relative changes to pressure gauge algometer readings**. (VAS: visual analog scale; PGA: pressure gauge algometer readings; Subocc: suboccipital trigger point location; Lev/Scap: levator scapulae insertion trigger point location; SCM: sternocleidomastoid trigger point location; TMJ: temporomandibular trigger point location).

**Figure 12:**
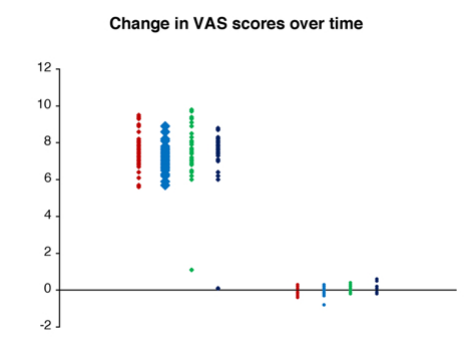
Scatter plot of individual changes to visual analog scale scores in both the control and treatment groups.

**Figure 13:**
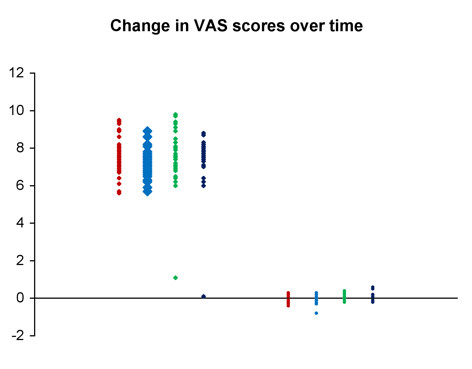
Scatter plot of individual changes to pressure gauge algometer readings for both control and treatment groups.

At all four trigger points, the average change in both VAS scores and PGA measurements for the treatment group was strongly significantly different (P < 0.001) from that for the control group. The mean changes for the two groups are presented in Table 3. In every case, there was strong evidence (P < 0.001) of unequal sample variances, and hence, for the t tests of equal means, variances were estimated separately for each group. (This form of the t test is sometimes called the Satterthwaite t test.) Relative changes to VAS and PGA have been shown in Figures 10 and 11. Scatter plots of raw data is presented for the reader to visualise the variation between individuals for both the VAS and PGA outcome measures (Fig 12 and 13).

**Table 3:** Changes to trigger point sensitivity in control and treatment groups.

		**CONTROL **(n = 20)		**TREATMENT **(n = 40)	
		
		Mean	SEM	Mean	SEM
**VAS**	Subocc	0.02	0.05	7.6	0.16
	Lev/Scap	-0.03	0.06	7.2	0.11
	SCM	0.14	0.04	7.6	0.24
	TMJ	0.13	0.07	7.3	0.28
**PGA**	Subocc	0.09	0.04	5.8	0.09
	Lev/Scap	0.05	0.05	5.8	0.08
	SCM	0.06	0.04	5.9	0.11
	TMJ	0.08	0.05	5.8	0.23

VAS: visual analog scale; PGA: pressure gauge algometer readings; Subocc: suboccipital trigger point location; Lev/Scap: levator scapulae insertion trigger point location; SCM: sternocleidomastoid trigger point location; TMJ: temporomandibular trigger point location)

### Control group

For the control group, the change in mean VAS scores was significantly different from 0 (P = 0.003) at the SCM trigger point. However, the difference, 14 mm, was only just clinically important; the 95% confidence interval (CI) for this difference was (5 mm, 22 mm). None of the other seven mean changes was significantly different from 0.

### Treatment group

For the treatment group, all eight individual changes were strongly significantly different from 0 (P < 0.001). The mean change in VAS scale for S was 7.6 (95% CI: 7.3, 7.9), LS was 7.2 (95% CI: 7.0, 7.4), SCM was 7.3 (95% CI: 6.5, 8.1) and TMJ was 6.9 (95% CI: 6.1, 7.7). The mean change in PGA readings for S was 5.8 (95% CI: 5.6, 6.0), LS was 5.8 (95% CI: 5.7, 6.0), SCM was 5.9 (95% CI: 5.7, 6.1) and TMJ was 5.8 (95% CI: 5.4, 6.3).

### Correlation coefficients

Improvement at one particular trigger point was not associated with similar improvements in the same subject for any other measurement-trigger point combinations. There were no significant pair-wise correlations amongst the eight improvements (i.e., both VAS and PGA measurements and the four trigger points; P values > 0.05; Pearson Correlation Test).

## Discussion

Recordings of visual analog scale significantly decreased in sensitivity, and pressure algometer readings significantly increased after a single NET treatment. In this cohort, the use of an NET based cognitive restructuring and meridian correction protocol, which purports to identify an initial causative issue of the presenting pain and dysfunction, had the ability to produce good improvements in simple muscle pain outcomes in the short term, in a small cohort of chronic neck pain sufferers. As a part of the NET protocol psychosocial components of the physical condition are considered. For example, emotion and memory associated with any relevant traumatic event is considered important in the recall of the painful experience, as investigated with the NET process. Participants are encouraged to reflect with direct referential statements on the variables whilst also considering the presenting symptoms. The technique does not incorporate a "talk it out therapy" or attempt to provide any psychoanalysis during the process.

Pain is a complicated, individual and variable experience [41]. Pain can alter in different conditions so it is important to assess pain under standardised conditions. Pain should be assessed: in the same location, on the same type of tissue (muscle) in a similar area of sensitivity (neck has a different sensitivity to a back or a knee), by the same practitioner using the same method of assessment [4,40,42]. Patient factors such as attitude, sex, cultural role and age must be recognised in studies such as this, in which participants record their own levels of pain [44].

Algometry is used to measure the sensitivity of pain or pressure [4]. Algometer instrumentation can include manual and electric models. The use of any experimental instrument including the PGA must be tested for validity and reliability between examiners and between performances of the same examiner. The PGA used in this study has been tested against itself, palpation, pressure plates with reliable results [17,18]. However, the above is based on the assumption that the tester is trained in the application of the PGA otherwise issues associated with the rate of pressure application [45-47] the determination of an end point based on a verbal patient response [45,46] and the possible sensitisation of a selected landmark based on repeated measures may all alter the accuracy of the outcome.

This study used the PGA in near optimal conditions. Chiropractor one, who used the PGA in the assessment of the trigger points had been routinely using the PGA for several years in a similar fashion to the use described in this study and was thus highly trained in the operation of the PGA. The electronic device, not used in this protocol, has been described as being superior to non-electronic algometric devices as such devices can control the rate of pressure application and minimise examiner reaction as well as calibrate itself [48]. The use of a highly trained individual minimised the risk of error from these sources but did not eliminate it [49]. Despite early support, the ongoing utilisation of pressure algometry in manual therapy warrants further research into the validity, effectiveness and best-use principles of such an instrument.

This study represented a first small step into creating a body of clinical literature on the usefulness of NET treatment in chronic neck pain patients with trigger point pain. The result of this study is encouraging for the management of trigger points and suggests psychosocial variables may have a beneficial effect on the intensity of trigger points. This view is supported by the work of others [50-53]. This contrasts with the usually described mechanisms of trigger points that are more local or spinal in nature [54,55]. However, if this research is reproducible it is still unknown which component of NET is useful for painful trigger points. It maybe that some, or all, components are useful.

### Limitations

No sample size was calculated prior to the commencement of this research project. Whilst the validity and reliability of NET treatments is far from proven, this study establishes data that may be used in power calculations of future studies to ensure that the sample sizes are large enough to detect a worthwhile and statistically significant effect.

No outcome measures were used to measure patient neck pain, neck disability or global overall improvement as the focus of this study was the resolution of the trigger points. It is recommended that future studies specifically include neck pain and a co variable and use appropriate outcome measures such as the Neck Disability Index [56] or the Patient-Specific Functional Scale [57]. In this trial we used patient perceived pain levels and the amount of sustainable pressure via algometer readings at the trigger point site to denote the level of activity of the trigger points. However, more extensive and clinically relevant outcome measures will be required for use in the interpretation of the effectiveness of this intervention in neck pain in the clinical setting.

With regard to the ratio of treatment to control participants, the 2:1 ratio of allocation was chosen for two pragmatic reasons. The first was to better estimate the effect of this preliminary investigation on the target tissues. The second and possibly more important factor, was the ethical requirement to provide patients who presented with pain the best treatment possible, and not a sham/control pseudo treatment. Future studies could incorporate a "waiting list" approach [58] to treatment rendered to the control group after allocation and completion of "treatment" rendered in the sham/control group and after appropriate informed consent.

This study was quasi-experimental; participants were sequentially allocated into treatment and control groups. Quasi-experiments are potentially prone to selection bias, that is, unobservable effects that are either unknown to the researcher or not easily measured which may ultimately affect the study outcome. Randomised controlled trials are the gold standard in evidence based research for efficacy and causal relationship, whilst quasi-experimental studies provide evidence for clinical effectiveness and generalisability of results. In contrast to the evidence supporting preferred use of RCT over quasi-experimental trial, two recent meta-analyses presented evidence that non-randomised trial data may not be inferior to that obtained from RCTs [59,60]. They concluded that the value of the trial rests largely on its real world validity, but this presupposes that the trial is till structurally sound as it would be with an RCT.

## Conclusion

Trigger points have been shown to be active in many myofascial pain syndromes, and previous to this study, the treatment of such trigger points supported the potential use of central and peripheral approaches to relieve pain and dysfunction associated with trigger points. Neuro Emotional Technique was administered to provide participants with a mind/body based treatment to relieve the sensitivity of trigger points associated with their chronic neck pain. It was found that after a short course of NET treatment, visual analog scale and pressure algometer measurements of four trigger point locations were significantly reduced compared to pre-treatment. A sham NET protocol did not produce significant changes in visual analog scale or pressure algometer measurements. The successful clinic based outcomes suggest that a mind body approach to the management of trigger points with NET should be considered in the management of trigger points in neck pain sufferers. Further evidence is required for better substantiation of the use in conservative management, with randomised controlled trials for the effect of NET on chronic neck pain, and other chronic pain syndromes recommended.

## List of abbreviations

NET: Neuro Emotional Technique; ICC: Interclass correlation co-efficient; S: Suboccipital; LS: Levator scapulae; SCM: Sternocleidomastoid; TMJ: temporomandibular region; VAS: Visual analog scale; PGA: Pressure gauge algometer; CAM: complementary and alternative medicine; ANOVA: Analysis of variance; SD: Standard deviation; CI: Confidence Interval.

## Competing interests

No funding was received in the preparation of this manuscript.

PB: Is a research student of Macquarie University.

HP: is a part time employee of 'The ONE (Our Net Effect) Research Foundation', a non-profit organisation. As an employee of this organization his interest in this research would relate to the foundation's mission statement: to establish natural healing as a standardised care through Neuro-Emotional Technique (NET) research, education and public service.

RB: No competing interests.

## Authors' contributions

* These authors contributed equally to this work

PB Conceived the idea of the study, wrote and edited the manuscript

HP Conceived the idea of the study, wrote and edited the manuscript.

RB Involved in data collection, reviewed and edited the manuscript

All authors read and approved the final manuscript.

## Supplementary Material

Additional file 1 NET Body Entry Protocol. The detailed description provided outlines the first 12 steps of the Neuro Emotional Technique protocol (adapted from Walker, 1996 [34]). Click here for file
