# Supplementary material for: Neuro Emotional Technique for the treatment of trigger point sensitivity in chronic neck pain sufferers: A controlled clinical trial
Source: Chiropr Osteopat. 2008 May 21;16:4. doi: 10.1186/1746-1340-16-4 (PMC2427032; doi:10.1186/1746-1340-16-4)
Supplement: Additional file 1 — NET Body Entry Protocol. The detailed description provided outlines the first 12 steps of the Neuro Emotional Technique protocol (adapted from Walker, 1996 [34]). [file 1746-1340-16-4-S1.pdf]

NET BODY-ENTRY PROTOCOL

Examine the region in lesion

A) Locate an active Trigger Point, a tender spot along a muscle within the lesion area. Perform a muscle test on a healthy muscle, other than the muscle with the identified trigger point. Note the general strength or tone of the muscle when lightly tested. Now the practitioner places their index finger on the identified trigger point and retests the muscle. If, on retest the muscle strength is now weaker then this indicates a so-called ‘body entry point’.

B) Establish if an emotional issue is involved in the pathogenesis of the trigger point that is being tested. The patient places their flat hand palm down across their forehead (The Emotional Point-Walker 1996) whilst the therapist maintains contact on the active trigger point with their index finger found in step 1. The Patient raises their other arm anterior palm down to 90% activating the anterior deltoid muscle. The practitioner with their other hand muscle tests the patient’s anterior deltoid by pushing down vertically at 90% on the patient’s outstretched arm. If this muscle test also remains weak as initially induced in step 1, then this protocol ceases as incorporating the contact on the forehead (emotional points) did not elicit a change in muscle strength. Thus no emotional issue involved in the pathogenesis of this particular trigger point that’s being contacted. If the muscle which, on previous testing became weak now tests as strong, then this indicates that a so-called ‘Neuroemotional Complex’ (NEC) has been identified. An NEC is a subjective maladaptation syndrome adopted by the human organism in response to a real or perceived stress. The individual’s unique NEC contains;

- A specific subluxation or sequence of subluxations.
- A specific emotion
- A conditioned response: A predisposition for stimulus generalisation. A resistance to extinction.
- A meridian imbalance and active pulse point.
- A facilitated or inhibited muscle.
- A specific active Meridian Access Point.
- A cathected and often recallable memory picture (Snapshot) of a past significant emotional event.

- A vulnerability to suppression, repetition compulsion and restimulation/reaggravation causing cyclical reinforcement. (Walker 1996).

The identification of a NEC is the primary indicator that NET treatment protocol is indicated in that patient for that particular trigger point.

Following the identification of the presence of a NEC the practitioner and patient remove all muscle, trigger point and forehead hand contacts and commence a specific delineated form of diagnostic dialogue which seeks to identify physical reactions to verbal stimuli.

Semantic muscle testing

Step 1: At this point semantic muscle testing is applied. The protocol of Walker 1996 is followed. The relevant semantic content of the NEC is testing by screening aberrant physical reactions (muscle strength) to three psychological domains termed-:

- “Money”, “Love” and “You”.

Once the main domain is determined by muscle testing then a further delineated methodical flow chart like approach is applied using verbal cues and semantic statements guided by the muscle test response until such a time that the core issue is arrived to. (Net Body-Entry Flowchart, Walker 1996).

Once a category “issue” is identified, a muscle which previously tested as strong, will become weaker on testing when the patient is challenged with the relevant semantic statement.

> Consider the following example; in a case where a patient’s muscle tested weaker after being asked to consider the concept of ‘love’, subsequent semantic challenges would follow to identify what aspect of ‘love’ was involved. For instance a subsequent semantic challenge may be the word ‘partner’ after which the muscle remained strong, indicating that love of a partner was not part of the emotional problem. A further challenge using the word ‘family’ may evoke a weakening of the muscle again. This would indicate that a family member (other than a partner) is perceived as part of the emotional problem. Further semantic challenges would ensue such as ‘mother’, ‘father’, ‘sister’, brother’, etc. Any such verbal cue which caused the muscle being tested to become weak again, would implicate that category or person as part of the problem as perceived by the

patient. This basic pattern is repeated and refined by the practitioner identifying persons, activities and events, all in the form of verbal cues which create a reaction in the muscle being tested.

Step 2 & 3: The next step involves Meridian Access Points (MAP's). MAP's are circumscribed points on the body that are representative of Acupuncture Alarm Points. They are points on the body that refer to specific organs and when contacting them you are accessing the organs via the cutis-visceral reflex. This step ascertains which organs alas which emotion is associated with the 'relevant' issue. The practitioner is to contact with his fingers all the MAP's in a sequential order whilst the patient continually thinks of/ contemplates the "relevant" issue. When the patient is contemplating the 'relevant' issue a healthy strong muscle will go weak due to the stressful neuronal afferrence from the limbic system and incongruent thoughts. When the practitioner is testing for the MAP that is associated with this particular 'relevant' issue a previously weak muscle test will go strong when contact is made on the correct MAP involved by the practitioner. MAP's are representative of the Acupuncture Organ meridians, as outlined by TCM-Traditional Chinese Medicine- alarm points (Walker 1996).

Eg: Once a NEC is located the practitioner seek to identify a link between that particular person or event and an emotion. To do this the practitioner touches key acupuncture points (MAP's), which in traditional Chinese medicine, are related to specific emotions. For instance, the gall bladder is said to be associated with anger and resentment, and this is reflected in English language metaphor when we talk of gall and bile in non-physiological contexts. If a patient with an identified NEC was found to have their muscle retest strong when the practitioner touched an acupuncture point (MAP), then that NEC would be said to be linked with the emotion associated with that acupuncture point (MAP). For instance, if contact with the kidney point caused the weakened muscle to retest strong, this would be an indication that the emotion of 'fear' was somehow involved in the establishment of the NEC.

The use of Acupuncture meridians and their related Emotions as correlated from traditional Chinese medicine is outlined in the NET manual published by this procedure's developer Walker in 1996.

Step 4: The next step is to ascertain whose emotion it is, namely whether the emotion is the patient's or someone else's acting on the patient. To do this the practitioner looks for the now strengthened muscle to go weak.

> In the above case, to evaluate whose emotion it was, the practitioner once again uses semantic challenges with statements such as - "Your fear X" or "someone else's fear X". In this instance if "Your fear" made the strong muscle go weak again this would indicate that it was the patient's own fear as an important factor in the development of the NEC.

Step 5: At this time the practitioner constructs a concept using the "relevant" issue and emotion thus far deduced. This concept is related to the patient and further expanded incorporating a search for a "*because"* or "*why"* component.

> For instance, the linking of the NEC which included 'father' and 'fear' would be explored.

The patient is asked to vocalise the present "relevant" issue with a "because" statement. A positive association will be demonstrated when a strong muscle becomes weak on vocalising the correct statement that is integral to the NEC.

> Eg: In constructing the concept the practitioner may inquire, "Why do you Fear with or about your father? The patient may reply "*because* he is very ill at present and I fear he might die!"

Step 6: The next step is designed to establish any associated Pavlovian conditioning. That is, an exploration of an original event in which a fear was perceived, and which may have continued to be expressed even after the removal of the fearful stressor. This is done by semantically assessing the concept of the original event when the emotion probably occurred (using Lowest common denominator – LCD, the common theme). The practitioner seeks to assess the concept of the original event where there was the emotion under investigation (eg fear) occurring linked to the 'because' statement also identified (eg because of serious illness).

In the protocol, the patient is now challenged with a series of semantic statements linking fear and serious illness in a loved one. Muscle testing is used so that if a strong muscle goes weak it would indicate an original event is adding to or instigating the stress/pain currently being experienced.

> Eg: In this case the practitioner may ask the patient … " The concept of an original event when you had *fear* for someone close to you because of their health or dying". An otherwise strong muscle testing weak on such a challenge indicates an original event said to be part of the NEC.

Step 7: The next step is to index the identified event for time. Indexing for time, occurs when the practitioner semantically challenges the patient for an original time, an age, in which a similar event had occurred that created similar distresses. The original event may involve a similar impact of that memory that had not been integrated and extinguished in the usual way when stressful events pass. The lack of extinguishing emotions from previous events is what gives rise to 'associated conditioned pavlovian responses' to current events that are burdened not only by the immediate stress at hand but also the distress experienced, complete with memory and affect retained (albeit subconsciously) .Indexing for time and age assists in getting to the original event, as outlined in the NET Manual by Walker 1996.

> Eg: When a strong muscle turns weak indicating an original event, the practitioner proceeds to the indexing for when this original event may have occurred with semantic muscle testing using the semantic statements " Conception to five years of age?", "six to ten years of age?", "eleven to fifteen", and so on. If, for instance, the patient's muscle became weak with the challenge of "six to ten years of age", the process is repeated using specific years with the following statements – "Six to seven years", "seven to eight years" and so on. If the strong muscle became weak with the semantic challenge for the age of seven, this would imply that the patient who was say 38 years old at the time of consultation responded to an event at age seven with semantic muscle testing.

Step 8: The practitioner attempts to facilitate the patient in finding the 'original event', in which in the above case appears to have occurred at the age of seven years. The

practitioner typically asks, “Where were you and what were you doing at the age of seven?” If the patient has trouble identifying the original event, the practitioner can help by testing possible categories using further semantic challenges with muscle tests. When the ‘original event’ is identified, a strong muscle tests weak.

> Eg: If our 38 year old the patient was asked ”Where were you and what were you doing at about seven years of age? Was anyone close to you or your family not well or dying?” The patient may respond as follows - that at age seven he heard that his Grandfather was diagnosed with cancer and that he constantly lived in fear of losing him every day. He subsequently did lose his grandfather three months later and that this affected him immensely as he was closer to him than his father. The patient now feared losing his father and reliving the same experience again.

Step 9: The practitioner attempts to correlate that the original issue now deduced in step 8 is associated and has the same MAP as found in step 2. Whilst the patient contemplates the original event, a healthy strong muscle will go weak on muscle testing. So as the patient contemplates the original event the practitioner performs a muscle test whilst also contacting the original MAP from step 2. If the weak muscle being tested by the practitioner goes strong whilst contacting this point, the same MAP applies for the original event deduced in step 8. This is usually the case. In rare occasions, if the muscle test does not become strong when contacting the original MAP from step 2 the practitioner is required to reassess for a new MAP and alas a new Emotion using the same protocol described above in steps 2 to 4. Then the rest of the procedure and protocol is followed through with the new MAP and Emotion referencing the original event.

> Eg: In this example, the MAP and Emotion deduced for the current situation were also the same for the original event. In this case, the right loin MAP caused the weak muscle to strengthen indicating that the Kidney Meridian for the Emotion of Fear was relevant to the trigger point pain presentation.

The correction

Step 10: The NET correction is applied through the application of three simultaneous actions. The correction is administered whilst the patient performs three discrete actions.

1) The Original event, (by contemplation and holding on the memory, feelings and emotions of the original experience).

2) The Emotional Points (by contacting and holding the forehead).

3) By contacting and holding the relevant MAP.

The correction itself involves the patient maintaining the contact points mentioned above whilst contemplating the original event whilst receiving a bilateral spinal stimulus in a specified spinal sequence. The patient holds the identified contact points whilst contemplating the original event whilst receiving stimulation from the practitioner at the specific sites outlined by the clinical NET manual during the 3 phases of respiration (inspiration, holding breath, expiration).

> Eg: in this case the patient held his forehead (Emotional Points) with his right hand and his right loin region (Kidney MAP) with his left hand whilst he contemplated the intense fear he was going through for 3 months about the possibility of losing his grandfather. As he held these points during contemplation, the practitioner applied bilateral spinal adjustments with mechanically assisted manipulation above the facets in a sequence indicated by the NET manual. This process is referred to as the "correction".

Step 11: After the "correction", the Original event is again contemplated whilst the practitioner retests the previously weak muscle (as described in step 8). A positive outcome is defined as a practitioner noting a strong muscle test after the contemplation of the original event (which prior to the correction caused the muscle test to test weak). If the muscle does not remain strong with contemplation of the original event then other emotions may also be present and steps 8 to 10 should be repeated until practitioner testing reveals a strong test following the contemplation of the original event.

Step 12: At this time, the practitioner should again perform a muscle test whilst the patient contacts the original problem area as described in Step 1 (in this case the trigger point). On testing by the practitioner, the muscle test of a strong muscle should remain strong whilst contacting the problem area if a correction has been completed. At this time, all cardinal signs of the trigger point should be removed.

Additional Steps 13, 14, 15: In the protocol described by the Walker manual include reassessing the active trigger point (the original Body Entry Point) for a Toxic BMI Combination with organ reflex, candidate remedy and then test re-test BMI Combination. The practitioner performs a muscle test on a strong muscle whilst the patient. The result of the practitioner muscle test demonstrated that the weak muscle corrected strong, the correct Remedy was performed. Finally, re-check at the Body Entry Point (Step 1) and the muscle tests strong and now stays strong, with resolution of the cardinal signs of the trigger point are resolved is possible. There should be no longer any pain associated with the trigger point on testing! The last steps are additional steps which complete the 15 step protocol as outlined by Walker 1996.
